# Pharmacological and toxicity effects of *Zhumeria majdae* and its bioactive constituents: A review

**DOI:** 10.22038/IJBMS.2022.64967.14304

**Published:** 2023-03

**Authors:** Kimia Khosravi, Arman Monajemi Mamaghani, Hossein Hosseinzadeh

**Affiliations:** 1 Department of Pharmacodynamics and Toxicology, School of Pharmacy, Mashhad University of Medical Sciences, Mashhad, Iran; 2 Pharmaceutical Research Center, Pharmaceutical Technology Institute, Mashhad University of Medical Sciences, Mashhad, Iran; # These authors contributed eqully to this work

**Keywords:** Analgesics, Anticonvulsants, Anti-infective agents, Anti-inflammatory agents, Camphor, Linalool, Morphine dependence, *Zhumeria majdae*

## Abstract

*Zhumeria majdae* Rech. F. & Wendelbo. traditionally has been used in several remedies, as a carminative agent especially for children, as an antiseptic agent, and it is used in treating diarrhea, stomach irritations, headaches, colds, convulsions, spasms, dysmenorrhea, and healing wounds. According to clinical studies, it is highly effective for reducing inflammation and pain, treating bacterial and fungal infections, morphine tolerance, morphine dependence, withdrawal syndrome symptoms, convulsions, and diabetes. The goal of this review is to find therapeutic opportunities by analyzing the traditional uses and pharmacological effects of the chemical constituents of *Z. majdae.*

The information on *Z. majdae* in this review was gathered from scientific databases or search engines (PubMed, Wiley Online Library, Scopus, SID, Google Scholar, and Microsoft Academic). The literature cited in this review dates from 1992 to 2021. Several bioactive components including linalool, camphor, manool, and bioactive diterpenoids are presen in different parts of *Z. majdae*. Various properties were observed such as antioxidant, antinociceptive, anti-inflammatory, antimicrobial, antiviral, larvicidal, anticonvulsant, antidiabetic, and anticancer properties. Also, the effect of *Z. majdae* on morphine tolerance, morphine dependence, and withdrawal syndrome as well as its toxicology has been established. Although there are *in vitro* and animal studies on several pharmacological effects of *Z. majdae*, the lack of clinical studies is significant. Therefore, further clinical trials should be performed to confirm the *in vitro* and animal findings.

## Introduction

*Zhumeria majdae* (also known as Mohrkhosh), the only species in the genus *Zhumeria, *is a fragrant perennial shrub that belongs to the *Lamiaceae* family and grows in some parts of Bandar Abbas, Hormozgan province, Iran (1). It was known in the past for its carminative property, especially for children. It is also known for its antiseptic activity and its uses for treating diarrhea, stomach irritation, headaches, colds, convulsions, spasms, dysmenorrhea, and healing wounds. The aerial parts of *Z. majdae* contain several pharmacologically useful compounds, such as linalool, camphor, and flavonoids (2, 3). As these compounds are recognized to be responsible for most of the effects of *Z. majdae* essential oil (ZMEO) which was known in the past, extraction and further purification might lead to the production of novel drugs that could be safer than their synthetic alternatives. In this paper, a list of all chemical constituents, their respective mechanisms and effects, and the associated pharmacological and toxicological data have been included based on a thorough review of existing *in vivo* and *in vitro* studies. However, it must be noted that there is a lack of clinical data to support the discovered effects of ZMEO as there are no human studies, all the studies in this review are either animal or *in vitro* microbial studies.

***Search Methodology***

In this article, we review the pharmacological effects of* Z. majdae *by evaluating studies without publication time limitations from different electronic databases or search engines such as PubMed, Scopus, Wiley Online Library, SID, Google Scholar, and Microsoft Academic. The following keywords were searched: “*Zhumeria majdae*,“ Mohrkhosh, constituents, “pharmacological effects,” anti-inflammatory, anti-tumor, cytotoxicity, antinociceptive, antimicrobial, antifungal, antibacterial, antiprotozoal, anticonvulsant, antidiabetic, antiviral, anti-oxidant, morphine tolerance, and morphine dependence. 68 articles were found without publication time limitation. The most relevant articles (31) which have evaluated the pharmacological effect of *Z. majdae* were included.

*Constituents*

**Figure 1:**
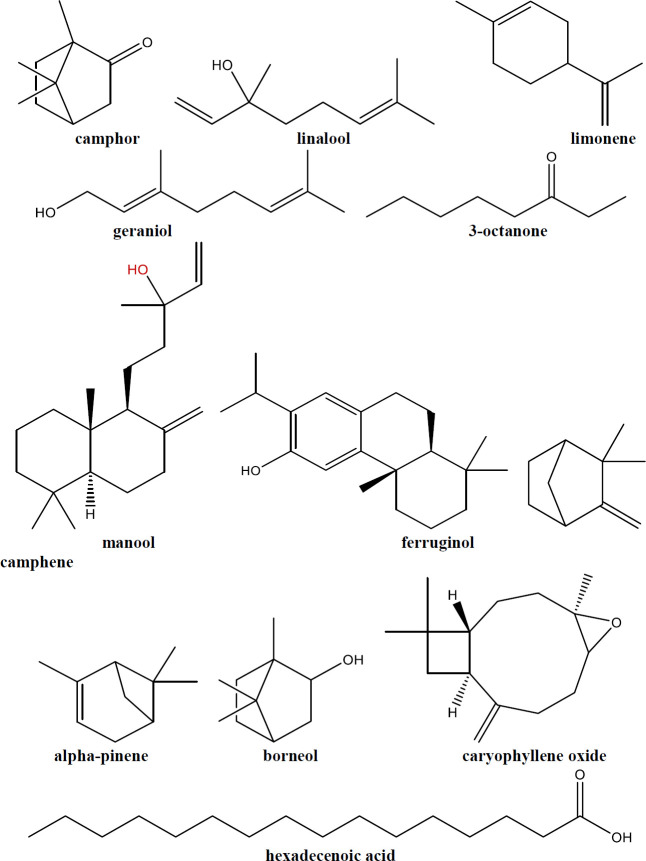
Main constituents of *Zhumeria majdae*

Using gas chromatography/mass spectrometry (GC/ MS) and GC, 17 compounds compose 99.13% of the ZMEO of aerial parts. The identified compounds and their abundance are as follows: linalool (63.4%), camphor (27.48%), trans-linalool oxide (1.11%), limonene (0.98%), geraniol (0.9%), borneol (0.83%), trans-linalool oxide (0.81%), p-menth-1-en-8-ol (0.63%), camphene (0.56%), 3-octanone (0.43%), caryophyllene oxide (0.40%), benzene, 1-methyl- 3-(1-methylethyl) (0.39%), Z-citral (0.32%), terpinen-4-ol (0.31%), geranial (0.28%), α-pinene (0.16%), and trans-caryophyllene (0.14%)(Figure 1). Linalool and camphor are found in various flowers and more than 200 spice plants, they are commonly industrialized due to their fragrant odor. In general, essential oils are complex mixtures of oxygenated mono- and sesquiterpene hydrocarbons. The total phenolic compound of ZMEO was also determined using the Folin-Ciocalteu reagent to be 42.74 GAE (mg)/ DW(g) (4).

ZMEO constituents were studied at different flowering stages in Geno, Sarchahan, and Tangezagh mountain areas in the Hormozgan province of Iran. Geno, Sarchahan, and Tangezagh, 7, 22, and 14 compounds were identified in the mountains, respectively. The main constituents were linalool and camphor in all three areas; however, the presence and the proportion of other constituents were different in each area. In the Sarchahan area, cis and trans linalool oxide, linalool, camphor, neral, geraniol, geranial, beta-elemene, and alpha-terpineol compounds were found, while in Tangezagh alpha-pinene, camphene, ortho cymene, limonene, borneol, nerol, and cis jasmone were identified, and in the Geno mountain gamma-terpinene, octa 3-carene, octan-3-one, myrcene, beta bisabolene, thymol, and terpinolene were the prominent compounds identified (5). Also, analysis of the root of *Z. majdae* showed the existence of two diterpene quinones with rearranged abietane skeletons, 12,16- dideoxy aegyptinone B and 12-deoxy-salvipisone, and manool (6). 

Another study has been done to find the composition of the oil of woody stems of *Z. majdae*. Results showed that manool was the main component (37.1%). After manool, most of the compositions belonged to hexadecanoic acid (3.5%) and trans-ferruginol (3.4%). Also, Linalool (2.1%) and camphor (0.8%) were observed in the stem oil (3). Refer to a study that examined the EO of the leaf of *Z. majdae,* eleven monoterpene hydrocarbons (13.8%), eight oxygenated monoterpenes (83.7%), and two sesquiterpenes (0.6%) were incorporated in the leaf EO. Camphor (42.1%), linalool (35.6%), camphene (4.1%), and limonene (3.4%) were the main components (7). 

*Pharmacological activities*

*Anti-inflammatory effects*

An *in vivo* study investigated the effect of aqueous infusion and ethanolic maceration extracts of the aerial parts of *Z. majdae* against acute and chronic inflammation. The anti-inflammatory properties of the extracts were studied using acetic acid which increased vascular permeability and xylol which caused ear edema in mice. Also, the extracts were tested for their effects against chronic inflammation using the cotton pellet test in rats. The extract and diclofenac were injected IP once daily for 7 days. The corresponding effects were similar to those of dexamethasone and diclofenac. In edema or acute inflammation of the ear in mice (triggered by xylol), unlike the aqueous extract, the ethanolic extracts of *Z. majdae* demonstrated anti-inflammatory activity. In small doses, the ethanolic extracts were as effective as diclofenac and dexamethasone. On the contrary, the aqueous extract was more effective when the acute inflammation was caused by acetic acid than the ethanolic extract (8).

Another *in vivo* study considers the anti-inflammatory effects of methanolic extracts of *Z. majdae* against edema caused by subcutaneous injection of carrageenan into the left hind paw of rats. The various doses of the extracts (100, 200, and 400 mg/kg) were administered via IP injection into the left hind paw. Paw edema in rats caused by carrageenan is used as a sensitive method for studying nonsteroidal anti-inflammatory agents. It shows a biphasic response which is attributed to several mediators. At the beginning (about 2 hr after injection of carrageenan), hyperemia is caused normally due to the release of histamine and serotonin, meanwhile, bradykinin and prostaglandins exacerbate swelling by inducing leukocyte mobilization. The results of this study demonstrated the anti-inflammatory effects of the methanolic extract (9). This phenomenon may be explained by referring to the presence of tannins and flavonoids in this plant. However, there is little evidence pointing to the anti-inflammatory activity of tannins. The extract inhibited the release of inflammatory mediators and potentially reduced capillary permeability (8).

Another *in vivo* study analyzes the effects of the EO extract from the leaves against acute inflammatory responses by carrageenan injection into the right hind paw of rats. In this study doses of 5, 10, 20, and 40 mg/kg of ZMEO were administered IP. The anti-inflammatory properties were similar to that of diclofenac sodium. It appears that the production of NO can lead to edema. ZMEO may reduce NO activity. The injection of carrageenan in rats causes bradykinin release which in turn leads to the biosynthesis of prostaglandin and other autacoids responsible for inflammatory responses. Linalool of EO can play a role in reducing inflammation by prostaglandin inhibition (10).

Although the above investigations proposed that *Z. majdae* was able to reduce inflammation, the lack of clinical studies was notable. So, further clinical trials should be accomplished to confirm the effect of *Z. majdae* on inflammation.

*Antinociceptive effects*

According to *in vivo* studies, the EO, aqueous, and ethanolic extracts of the aerial parts and the EO of the leaves of *Z. majdae* show dose-dependent central and peripheral antinociceptive effects in mice, proven using the hot-plate and acetic acid-induced writhing tests (8, 10). In the hot plate test, the IP injection of ethanolic and aqueous extracts both showed notable antinociceptive effects dose-dependently in mice. Pretreatment with naloxone, an opioid antagonist, can inhibit the antinociceptive effects of the extracts. The extracts also showed antinociceptive properties against acetic acid-induced writhing, which could also be inhibited by naloxone. These extracts can be as effective as diclofenac in antinociceptive properties (8). In the hot plate test, the effects of methanol extracts were most prominent at the doses of 200 and 400 mg/kg, significantly increasing the pain threshold and tolerance in mice (9). It was shown that antinociceptive effects might be mediated by the presence of flavonoids and tannins. Flavonoids such as hesperidin, luteolin, quercetin, rutin, and bioflavonoids have antinociceptive properties (8). This effect may also be caused by linalool as one of the main constituents of the extracts and EO. It was apparent that methanolic extracts have a stronger antinociceptive effect than aqueous extracts because linalool is more soluble in methanol than in water (9). The analgesic effects are caused by a decrease in voltage-dependent Na^+ ^flow in ganglion cells (11)10, 20, and 40 mg/kg intraperitoneally (i.p.. Camphor is another main constituent that could be responsible for such effects. It blocks TRPA1 and activates TRPV1 (12). ZMEO inhibits the prostaglandin synthesis pathway through the cyclooxygenase pathway, thereby causing antinociceptive effects. Aside from this, central and peripheral responses have been observed, pointing towards a unique biphasic licking response (10). The antinociceptive effects of the extract could also be mediated by central opioid receptors and/or the release of endogenous opioid peptides. Spinal opioid receptors may also be involved (8). 

*Antimicrobial activities*

*Antifungal effect*

In an *in vitro* study, the hydro-distilled oil of the aerial parts of *Z. majdae* has been examined for its antifungal activity using the punch well/cup plate diffusion method. Through serial dilution, the ZMEO was prepared at 0.007, 0.015, 0.031, 0.062, 0.125, 0.25, 0.5, 1, 2, and 4 μg/ml concentrations. The fungi were grown in Sabouraud’s dextrose broth. Inhibition zones were evaluated using a film disc containing antifungal agents. The MIC was measured using the serial dilution method. It demonstrated the potential for antifungal activity against pathogenic fungi such as *Candida albicans, Trichophyton mentagrophytes, Aspergillus flavus, Trichophyton rubrum, Microsporum canis, Microsporum gypseum*, and *Epidermophyton floccosum*. (Inhibition zone=7.84-29.05 mM , average MIC value=0.015 μl/ml). Results showed that ZMEO has the most antifungal effect on *C. albicans* with an inhibition zone diameter of 29.05 mm and a MIC of 0.031 μl/ml. ZMEO showed the least antifungal effect against *A. flavus* with an inhibition zone diameter of 7.84 mm and a MIC of 0.25 μl/ml (4).

In an *in vitro* study, the antifungal properties of ZMEO were determined using disc diffusion and micro broth dilution assay. Its potency was compared with that of amphotericin B against *C. albicans, A. flavus*, and* A. niger*. The growth medium for these fungi was Sabouraud’s dextrose agar. According to the results, the inhibition zone caused by 2.5 μl of the oil was smaller than the one caused by amphotericin B, with diameters of 10 and 17 mm, respectively. It was also effective against *A. flavus* and* A. niger*, however, it was less effective compared with its effects against *C. albicans*. *A. niger* was the most resistant toward ZMEO among the three species. The MIC and MBC values are as follows: 

*A. niger*: MIC= 0.5 μl/ml MBC=8 μl/ml

*A. flavus*: MIC=2 μl/ml MBC=8 μl/ml

*C. albicans*: MIC=2 μl/ml MBC=4 μl/ml (13).

Using disc diffusion, ZMEO was also found to have inhibitory effects on *Saccharomyces cerevisiae.* This fungus was cultivated along with *C. albicans* and *C. niger* in Sabourad’s dextrose agar plates with 10 μl of ZMEO. EO showed little antifungal activity, *S. cerevisiae *was the most sensitive to the oil among the samples with an inhibition zone of 16 mm and MIC of 5 mg/ml (14).

In another *in vitro* study, the antifungal activities of ZMEO against phytopathogenic fungi such as *Fusarium graminearum, F. asiaticum, F. redolens *f.sp.* dianthus, A. flavus, A. tubingensis, F. verticillioides, F. oxysporum *f.sp.* lentis, Botrytis cinerea, Sclerotinia sclerotiorum, and Cladosporium cladosporioides *were evaluated*. *The fungi were cultivated in PDA and slant synthetic nutrient agar media. *In vitro *analysis of the antifungal activity was performed in a PDA medium adding ZMEO at 75, 150, 300, 600, 1000, and 1500 μl/l concentrations. The oil had no effects (%0) at 75, 150, 300, and 600 μl/l, but showed full potency (100%) at 1500 μl/l against *F. graminearum, F. asiaticum, S. sclerotiorum.* *A. flavus* was the most resistant to ZMEO (15). 

Generally, terpenes and flavonoids are the main components in EO causing its antifungal properties. These properties may also be due to the presence of linalool and camphor in the oil. Knowing such properties may be useful in the pharmaceutical and cosmetic industries (4).

The encapsulated form of EO using lecithin (EFEO) demonstrated antifungal effects against *C. albicans* and *A. niger* when grown in Sabouraud dextrose agar medium. To compare the effects of EO and EFEO an* in vitro *study was conducted and the microdilution broth method was used at 0.6%, 1.25%, 2.5%, 5%, and 10% concentrations. 3 µg/ml of nystatin was used as the positive control. Results showed that 100 μl of EO and EFEO were equally effective against *A. niger* (MIC_50_ value of 0.6%, MIC_90_ value of 2.5% v/v). Against *C. albicans*, both forms had an MIC_50_ value of 0.6% v/v, however, the MIC_90_ value in the case of EO was 1.25% v/v, compared with 5% v/v in the case of EFEO; therefore, EFEO was more effective against *C. albicans* than EO, meaning encapsulation improved the antifungal property (16).

Another *in vitro* study investigated the inhibitory effect of the hydroalcoholic extract of *Z. majdae* leaves on tyrosinase in fungi. Inhibiting tyrosinase could play a role in the prevention and treatment of pigmentation diseases. In this study, 50 μl quantities of the *Z. majdae* hydroalcoholic extract were used at 0.05, 0.10, 0.21, 0.42, 0.83, and 1.67 mg/ml concentrations. After incubation, l-DOPA was added to the mix. Dopachrome was measured using the microplate reader to determine the degree of inhibition. Kojic acid was used as a positive control. Kinetics parameters of tyrosinase such as maximum velocity (V_m_) and Michaelis constant (K_m_) were determined via the Lineweaver-Burk plot. The extract at 1.67 mg/ml inhibited l-DOPA oxidation by 29.70%. The results showed that *Z. majdae* was weaker at inhibiting tyrosinase than kojic acid. The effects were dose-dependent and tyrosinase inhibition increased at higher extract concentrations. *Z. majdae* extracts decreased V_m_ and K_m_ of the fungal tyrosinase, whereas kojic acid decreased V_m_ but increased the K_m_ value; therefore, it can be concluded that *Z. majdae* extracts can only bind with the enzyme-substrate complex, while kojic acid can bind with the free enzyme and the enzyme-substrate complex alike. Antityrosinase properties of *Z. majdae* could be due to its flavonoid content. Besides flavonoids, *Z. majdae* extracts also contain anti-oxidants and free radical scavengers which cause antityrosinase activity (17).

Generally, the potency of antimicrobial effects of ZMEO varied in different circumstances, depending on the microorganism being tested and the lab environment, ranging from inhibitory to germicidal effects. Notably, the oil acts differently on spores from germination cells (13).

*Antiprotozoal effects*

Diseases such as American trypanosomiasis, human African trypanosomiasis, leishmaniasis, and malaria are some of the prominent causes of death worldwide. Due to the limited efficiency and side effects of the drugs currently in use and the risk of resistance against them, searching for new ways of treatment in this area is highly important, especially if the active ingredients may be retrieved from natural sources (18).

*Z. majdae* acts as a natural antiprotozoal agent against these parasites. Hexane, ethyl acetate, and methanol extracts of the root and aerial parts of *Z. majdae* have been carried out, *in vitro,* against *Trypanosoma brucei, Leishmania donovani, T. cruzi, T. brucei rhodesiense, and Plasmodium falciparum*. The extracts showed antiprotozoal activity, among which the root parts were the most potent. The hexane extract of the roots of *Z. majdae* was highly effective against *T. b. Rhodesiense* (IC_50_ value of 5.4 μg/ml), *P. falciparum* (IC_50_ value of 2.1 μg/ml), and *L. donovani* (IC_50_ value of 1.6 μg/ml). Antiprotozoal agents were isolated and identified using chromatography and spectroscopy. Eight compounds were recognized: triene (1), 12, 16-dideoxy aegyptinone B (2), ferruginol (3), Sugiol (4), Δ9-ferruginol (5), 11,14-dihydroxy-8,11,13-abietatrien-7-one (6), manool (7), and lanugon Q (8). These compounds have all shown antiprotozoal activity against *T. b. Rhodesiense* and *P. falciparum*, while showing no significant effects against *T. cruzi* and *L. donovani*. Among the compounds mentioned, the 6th and 8th compounds were the most active, their most significant effects were observed against *T. b. Rhodesiense* with IC_50_ values of 1.82 and 0.13 μM, respectively. The sixth compound showed the highest level of antiplasmodial activity relative to others. The 3rd and 5th compounds showed promising antiplasmodial properties with an IC_50_ value of 0.9 μM. According to the results of this study, it may be inferred that the higher antiparasitic activity of the roots compared with that of the aerial parts could be due to the presence of the 6th compound as an abieta-8,11,13-triene derivative and the 8th compound as a tanshinone-type diterpenoid (18).

ZMEO is effective against *T. b. Rhodesiense* and *P. falciparum* due to the presence of dihydroxy-8,11,13-abietatrien-7-one and lanugon Q, with higher selectivity in *T. b. Rhodesiense*. Another constituent, 12, 16-dideoxy aegyptinone, was potent against *T. b. Rhodesiense*, but ineffective against *P. falciparum* (18).

According to a study of the roots of *Z. majdae*, a terpene called 12,16-dideoxy aegyptinone B was found to be effective in the antiplasmodial and antileishmanial properties of *Z. majdae* extracts. Also, the antileishmanial activity of the methanolic extracts of *Z. majdae* has been evaluated *in vitro *via Alamar blue assay on *L. donovani* using pentamidine and amphotericin B as standard. The extracts demonstrated potent effects against leishmanial growth with an IC_50_ value of 1.1 μg/ml. 12,16-Dideoxy aegyptinone B with an IC_50_ value of 0.75 μg/ml was proven to be more potent than pentamidine, but less effective than amphotericin B. Antiplasmodial effects of the methanolic extract were analyzed by measuring the parasitic lactate dehydrogenase activity against chloroquine-sensitive and chloroquine-resistant strains of *P. falciparum*. The extracts showed potent antiplasmodial effects against chloroquine-sensitive strains of *P. falciparum* with an IC_50_ value of 8.8 μg/ml and chloroquine-resistant strains with an IC_50_ value of 7.5 μg/ml. 12,16-Dideoxy aegyptinone B also showed antiplasmodial activity against chloroquine-sensitive and chloroquine-resistant strains with IC_50_ values of 1.3 and 1.4 μg/ml, respectively. These values were inferior to those of pentamidine and amphotericin B (19). 

Further research is required in this area to elaborate on the mechanisms for the antileishmanial and antiplasmodial properties of *Z. majdae*.

*Antibacterial effects*

Antibiotic resistance of some pathogens along with harmful side effects of antibiotics has led to a rise in interest in finding new antimicrobial agents among herbal plants that are safe and more efficient (13). 

An *in vitro* study showed that methanol and chloroform extracts of aerial parts of *Z. majdae* and its EO have inhibitory effects on the suspension of *H. pylori* cells using the disc diffusion method. The inhibitory effects of *Z. majdae *extracts on *H. pylo*ri are as efficient as antibacterial agents, such as levofloxacin, clarithromycin, and metronidazole**.** The inhibitory effects were most significant at 50 mg/ml concentration in methanol and chloroform extracts. EO shows antimicrobial properties against *H. pylori* at 1/10 and 2/20 concentrations. Overall, ZMEO is more effective against *H. pylori* than methanol and chloroform extracts (20)**.**

Besides that, *in vitro* assessment through disc diffusion and micro broth dilution assay has proven the antibacterial effects of ZMEO against *Staphylococcus aureus, S. epidermidis, S. saprophyticus, Listeria monocytogenes, Bacillus cereus, B. subtilis, B. pumilus, E. coli, Salmonella typhi, Pseudomonas aeruginosa, Shigella dysenteriae, S. flexneri, Klebsiella pneumoniae, Proteus vulgaris, Enterobacter aerogenes, *and* Enterococcus faecalis.* This was tested using plates containing the suspension of the bacteria in Mueller Hinton agar (13). Among bacterial organisms such as* S. aureus, E. faecalis, B. subtilis, K. pneumoniae, P. aeruginosa, E. coli, S. epidermidis, *and* B. pumilus*, three of which are the most sensitive to ZMEO, *B. subtilis, B. pumilus, *and *S. epidermidis*, with inhibition zones of 35, 34, and 31 mm, and MIC values of 0.93, 0.93, and 1.87 mg/ml, respectively. *P. aeruginosa* was determined to be the most resistant to ZMEO. Generally, ZMEO highly inhibits Gram-positive bacteria (except for *E. faecalis*), however, its effects range from low to moderate on Gram-negative bacteria (14). ZMEO at doses of 0.0125-8 μm/ml produces an inhibition zone of 2.5 μm in the case of Gram-positive bacteria such as *S. aureus, S. epidermidis, *and* S. saprophyticus*, which is smaller than that of vancomycin. In the case of *L. monocytogenes, B. cereus*, and* B. subtilis*, the inhibition zone is smaller than when erythromycin is used, among Gram-negative bacteria, *E. coli, S. Typhi, P. aeruginosa, S. dysantriae, S. flexeneri, K. pneumoniae, P. vulgaris, E. aerogenes*, and *Vibrio cholera*, the inhibition zone is smaller compared with when gentamicin is used. Among these, *S. typhi *and* P. aeruginosa* from Gram-negative bacteria, and *B. subtilis* from Gram-positive bacteria were the most resistant to ZMEO. *K. pneumoniae* was determined to be the most sensitive species, with MIC and MBC values of 0.5 and 1 μl/ml, respectively. *S. aureus* came second in sensitivity (MIC=1 μl/ml, MBC=1 μl/ml). Next came *V. cholera* and *B. cereus *both with MIC and MBC of 2 μl/ml (13)**. **ZMEO showed bactericidal effects against *S. aureus, B. cereus,* *E. aerogenes*, and *E. coli* with the following MIC and MBC values: 

*S. aureus*: MIC=1 μl/ml, MBC=1 μl/ml;* B. cereus*: MIC=2 μl/ml, MBC=2 μl/ml; *E. aerugenes*: MIC=4 μl/ml, MBC=4 μl/ml; *E. coli*: MIC=4 μl/ml, MBC=4 μl/ml (13).

An *in vitro* study uses hole-plate diffusion to evaluate the antibacterial properties of ZMEO, in which a homogenous suspension of bacteria cells was grown in a Mueller-Hinton agar culture. At different concentrations, the EO, aqueous, and methanol extracts were tested against several bacteria, including *S. aureus, E. coli, K. pneumoniae, S. epidermidis, B. subtilis, *and* P. aeruginosa*. ZMEO at 0.39 mg/ml showed a notable antibacterial activity. It was also discovered that the petroleum ether extract is effective against some strains as well. The antibacterial spectrum of this type of extract is similar to that of EO; however, the lower potency, probably means they share some similar compounds. *Z. majdae* methanol extract is most efficient against *P. aeruginosa*. This activity may be mostly due to the presence of flavonoids, polar thermo-labile, and/or thermo-stable phenolics (21).

Generally, Gram-negative bacteria tend to show more resistance against ZMEO than Gram-positive bacteria, due to the presence of lipopolysaccharide in their outer membranes (13).

The EFEO by lecithin was determined *in vitro* to be effective against *E. coli* and *S. aureus* by measuring MIC value using the microdilution broth technique. The cells were first cultivated in nutrient agar, then they were each added to Mueller Hinton broth. 5 µg/ml of ciprofloxacin was used as a positive control. ZMEO and EFEO showed equal MIC_50_ and MIC_90_ values of 0.6% and 1.25% v/v, respectively (16).

An *in vitro* microplate-based assay showed that ZMEO dose-dependently inhibited an antibiotic-sensitive strain of *E. coli *(Dh5α) growth. At 1:200 dilutions, the growth of *E. coli* was inhibited while an LC_50 _value occurred at 1:1000 dilutions (22). ZMEO contains substantial amounts of linalool and camphor with antibacterial properties. Also, the antibacterial effects of the crude extracts may be due to the presence of various phytochemical compounds. Linalool could have protein denaturing or solvent-dehydrating effects, which could play a role in the antibacterial activities of the oil (13). In reality, the antibacterial properties of ZMEO might be due to synergy, antagonism, or additive effects of various oil constituents (22). Therefore, further studies are necessary to confirm the exact antibacterial mechanism of *Z. majdae. *

***Antiviral effects***

The antiviral effects of the methanolic extract of *Z. majdae* have been tested *in vitro* on Vero cells against HSV-1 using plaque reduction assay. Acyclovir was used as a positive control. The result was inhibition of plaque formation for a period of 3 hr after infection at 5, 10, 20, and 50 μg/ml concentrations. This means that *Z. majdae* extracts show a high anti-HSV-1 effect at low concentrations. Rosmarinic acid probably plays a role in this effect, it has antiviral properties, and it accounts for 1.3% of the extract. Although rosmarinic acid is conclusively effective in this case, other constituents such as flavonoids, derivatives of caffeic acid, and tannins could also contribute to this effect by blocking the surface ligands and receptors of either the virus or the host (23).

***Insecticidal effects***

Mosquitos are a significant method of spreading diseases. Malaria is a serious blood disease caused by *plasmodium* parasites invading the red blood cells. It is transmitted through the bite of the *Anopheles* mosquito. *Culex quinquefasciatus* is a common vector that acts as a regional vector of *Wuchereria bancrofti* and a possible vector of the West Nile virus. An *in vitro* study demonstrates that ZMEO extracted from the leaves can eradicate larvae of *Anopheles stephensi* and *Culex quinquefasciatus*. Lethal concentration values for these species are *A. stephensi* LC_50_=61.3 ppm, LC_90_=135.8 ppm; *C. quinquefasciatus* LC_50_=88.51 ppm, LC_90_=191.56 ppm (24).

The comparison of the regression coefficient in two mosquito populations proved that *An. stephensi* larvae were destroyed highly dose-dependently. ZMEO influenced *An. stephensi* larvae more than the larvae of *Cx. quinquefasciatus*, which could be related to genetic and physiological differences between the two species (24). 

***Cytotoxic effects***

The cytotoxic activity of the methanolic extracts of *Z. majdae* roots along with its diterpene content was studied *in vitro* in a panel of cancer cell lines. Cytotoxicity against mammalian fibroblasts and macrophages and the toxicity against the panel of cancer cell lines have been analyzed. The given cell lines included human malignant melanoma (SK-MEL), human epidermal carcinoma (strain KB), human ductal carcinoma (BT549), human ovary carcinoma (SK-OV3), monkey kidney fibroblast (Vero), and mouse macrophages (RAW 264.7). After 48 hr of incubation, the cytotoxic activity was determined to be between 50 to 80 μg/ml for the methanolic extracts and 4.5 to 15 μg/ml for the diterpene compound, namely, dideoxy aegyptinone B-12, 16 present in *Z. majdae*. In comparison with doxorubicin, the diterpene compound in *Z. majdae* has much lower toxicity against mammalian cells (19).

In another *in vitro* study, the cytotoxicity of ZMEO on two human cancer cell lines has been investigated by using an MTT assay. EOs were extracted from aerial parts of *Z. majdae* in its flowering stage at five places in the south of Iran: Sirmand; S1, Ghotbabad; S2, Sarchahan; S3, Tangezagh; S4, and Geno; S5. Rising doses of ZMEO in the concentration range of 12 to 1600 μg/ml were exposed to the two human cancer cell lines; A375 (human melanoma cell line) and MCF7 (human breast cancer cell line) for 48 hr. Their growth was inhibited concentration-dependently. The A375 cell line growth was inhibited by S1, S2, S3, S4, and S5 EOs with IC_50_ values of 746.6, 666.1, 624.6, 779.5, and 718.8 μg/ml, respectively. The MCF7 cell line growth was inhibited by S1, S2, S3, S4, and S5 EOs with IC_50_ values of 674.3, 717.9, 732.3, 646.9, and 642.7 μg/ml, respectively. There were no significant differences between the cytotoxicity properties of EOs in the different areas, all having moderate cytotoxicity. More precisely, S3 and S5 had the most potent effects on A375 and MCF7, respectively. Camphor and linalool were possibly responsible for the cytotoxic activity. Linalool induces cells to undergo apoptosis and causes cell death. Various minor constituents may also cause a synergistic effect leading to more cytotoxicity. Further research is necessary to determine the underlying mechanism and confirm its safety (25).

One recent *in vitro* study investigated the antiproliferative effect of hexane extract of *Z. majdae* roots against HeLa (Human cervix epithelial carcinoma) and MCF7 (human breast adenocarcinoma) cell lines by evaluating the binding affinity of compounds isolated from *Z. majdae* roots to HSP90 protein. HSP90 proteins react to cellular stress and play a major role in anti-apoptotic proliferative processes. 21 diterpenoids were isolated from hexane extract. Using surface plasmon resonance, the inhibitory effect of these diterpenoids on HSP90 protein was evaluated. Results showed that 8 out of 21 diterpenoids can bind to HSP90 α. Another constituent, lanugon Q, had a great affinity for the chaperone, with a KD value of 2.97±0.5 nM. Also, this compound at the concentration of 0.4-60.0 μM showed the anti-tumor effect in MCF7 cell lines with an IC_50_ value=20±2 μM by MTT assay. None of these compounds were effective against HeLa cell lines. In addition, the proliferation of PBMC (non-tumor human peripheral blood mononuclear cell line) was not affected by any of the compounds due to the IC_50_ value of cytotoxicity activity being up to 100 μM. As examined using western blot, lanugon Q reduced the intracellular concentration of HSP90-dependent client proteins, cyclin A, p-Akt, and p-Erk. Due to their role in anti-apoptotic and proliferative pathways of cancer cells, a decrease in their concentration can eventually lead to the inhibition of cancer cell proliferation. The mechanism of action of lanugon Q still needs to be elaborated, especially the way the effects differ *in vitro* compared with *in vivo* (26). Further research on various other cancer cell lines and different cell growth factors is necessary to confirm the antiproliferative property of *Z. majdae*.

***Anticonvulsant effects***

**In an** *in vivo* study, the IP injection of ZMEO, 60 min before inducing seizure, demonstrated anticonvulsant activity in PTZ and MES models in mice, protecting them from tonic convulsions (at an effective dose (ED_50_) of 0.25 ml/kg in PTZ and 0.27 ml/kg in MES). This effect is most probably due to the presence of linalool (which also causes sedation) and other terpenoids (27). Linalool is known to have inhibitory effects on nervous excitability, as tested in various mouse seizure models (28). Borneol is another constituent of ZMEO which could be responsible for its anticonvulsant properties (29). ZMEO significantly increased the onset of tonic convulsions, with the peak at 0.25 μl/kg in mice PTZ models. While the measured lethal dose (LD) was much higher than the anticonvulsant dose (LD_50_ = 2.35 vs ED_50_ = 0.27 ml/kg), it might still cause neurotoxicity. At doses close to the seizure dose (TD50 of 0.55 ml/kg) motor deficit and sedation arose. The therapeutic index (TI) of seizures from MES was 8.7 and the TI of PTZ seizures was 9.1. Therefore, ZMEO may be useful for its therapeutic effects. Lethality was linked to the presence of camphor, α,β-thujone, and camphene at high concentrations. In both PTZ and MES models in mice, the methanolic extract of the plant did not demonstrate anticonvulsant activity, except for dose-dependently increasing the onset of tonic convulsions in the PTZ model. This is due to the terpenoids being nonpolar which makes their extraction using methanol less effective (27). At a 2 kg/g dose, the methanolic extract significantly impaired motor coordination. The mortality rate at 5 g/kg was 34%. ZMEO and the methanolic extracts significantly decreased rotarod duration dose-dependently, at respective doses of 0.65 ml/kg and 2 g/kg (27). 

The frequency of clonic convulsions induced by PTZ IP injection was reduced at all doses of ZMEO (5, 10, 20, and 40 mg/kg). Along with increasing the onset, the administration of 20 mg/kg ZMEO significantly increased the chronic seizure threshold and lowered the mortality rate. These effects were comparable with diazepam. Co-treatment of ZMEO at 5 mg/kg with diazepam at 25 μg/kg prevented death in all mice that received IP administration of PTZ. Coupling these two compounds leads to a synergistic effect of increasing the latency and reducing the frequency of convulsions (11)10, 20, and 40 mg/kg intraperitoneally (i.p..

The protective properties of ZMEO against hindlimb tonic extensions (HLTEs) from MES testing were also analyzed. ZMEO could not protect the mice against HLTEs and death (at 5, 10, 20, and 40 mg/kg doses) (11)10, 20, and 40 mg/kg intraperitoneally (i.p.).

Under the PTZ-induced seizure model with IV injection, IP pretreatment with L-arginine (30 mg/kg) increased the protective properties of the oil by raising the clonic seizure threshold. Meanwhile, IP pretreatment with L-NAME (2.5 mg/kg) or aminoguanidine (100 mg/kg) reduced the protective properties of ZMEO and lowered the clonic seizure threshold (11)10, 20, and 40 mg/kg i.p.

After IP administration, ZMEO freely passes the blood-brain barrier due to being small and highly soluble in lipids. Stimulatory effects on GABA receptors are involved in the anticonvulsant activity of ZMEO. There are two types of GABA receptors, GABA_A_ and GABA_B_. The effects mentioned are according to studies involving pretreatment with flumazenil, an antagonist at the benzodiazepine binding site on the GABA_A_ receptor (11)10, 20, and 40 mg/kg i.p.

NO release is also an important factor. L-arginine, prescribed with the right dosage, raises NO levels which indirectly increases GABA-covered surface by inducing GABA secretion from various areas in the brain and inhibiting GABA aminotransferase activity. Therefore, it could be hypothesized that pretreatment with ZMEO causes NO up-regulation and protects the central nervous system from the seizure-inducing effects of gradual PTZ. L-NAME and aminoguanidine are also NOS inhibitors and prevent the synthesis of NO; therefore, at low concentrations, not enough to have any effects on the seizure threshold, these two inhibitors can completely reverse the anticonvulsant effect of ZMEO. Overall, the ZMEO derived from the aerial parts causes a biphasic anticonvulsive reaction in intraperitoneal (IP) and intravenous (IV) models of PTZ-induced seizure, significantly increasing the survival rate of the mice. However, under the MES model, ZMEO was relatively ineffective in raising the chances of survival in mice. On the other hand, it showed good potential against myoclonic or generalized absence epilepsy symptoms. This is especially important since absence seizures are growing resistant to available drugs (11) 10, 20, and 40 mg/kg i.p.

*Effects on morphine tolerance and dependence*

According to an *in vivo* assessment in mice, the EO extracted from *Z. majdae* leaves showed significant effects on morphine dependence and morphine tolerance. The dependence effect was evaluated by counting the number of jumps induced by naloxone (5 mg/kg) while the tolerance was assessed by the tail-flick test. In the model of morphine tolerance and dependence in mice, a repeated-injection schedule was used. Morphine was administered three times a day for three days for the dependence model, and for the tolerance model, it was injected twice a day for three days. ZMEO doses were injected 30 min before morphine injection for three days. It has been shown that 10 mg/kg of ZMEO can significantly reduce morphine tolerance. At the doses of 20 and 40 mg/kg, morphine dependence was reduced. The efficacy is similar to that of clonidine. Therefore, ZMEO is a good candidate to be used in the treatment of dependence and tolerance to morphine and other opioids in substance abuse patients (30).

An *in vivo* experiment used the tail-flick test on mice pretreated for four days with subcutaneous morphine (doses of 50, 50, 75, and 50 mg/kg from day one to day four, respectively), IP administration of various *Z. majdae* extracts at doses of 0.28, 1.12, and 1.96 g/kg, showed to have beneficial effects on morphine withdrawal syndrome. Naloxone at a 5 mg/kg dose was injected subcutaneously to cause withdrawal syndrome in mice. The IP injection of the extracts reduced withdrawal syndrome symptoms dose-dependently at all doses. The petroleum ether extract reduced the number of tail flicks just like the complete extract. This effect was determined to be dose-dependent, with the percentages of reduction at 0.28, 1.12, and 1.96 g/kg, being 74.8%, 93%, and 97.5%, respectively. The aqueous methanolic extract at all doses reduced the tail flicks dose-dependently as well, with the percentages of reduction at 0.28, 1.12, and 1.96 g/kg, being 64%, 83.7%, and 96%, respectively. The chloroform fraction at the highest dose showed significant effects compared with the negative control group. The percentage decrease in the number of tail flicks at 0.28 g/kg was 4.6%, at 1.12 g/kg, 10.9%, and at 1.96 g/kg, 18%. The MPLC column extracted fractions decreased the number of tail flicks in the withdrawal syndrome. The percentage decreases for A, B, and C fractions were 97.4%, 92.9%, and 55%, respectively. Between different fractions and extracts of *Z. majdae*, the chloroform fraction showed the least potency. The total methanolic extract significantly reduced withdrawal symptoms. The effects of the extract at a 1.96 g/kg dose were similar to the effects of diazepam at 5 g/kg, reducing movement and muscle contraction in mice (31). Linalool, monoterpene (in high proportions), limonene, and alpha-pinene found in *Z. majdae* are responsible for muscle relaxation and the decrease in tonicity by affecting the cAMP system (32). Fractions A and B showed the same potency as diazepam in reducing the number of tail flicks and recovering from withdrawal. At similar doses, the aqueous methanolic extracts have a greater impact than the chloroform fraction. Meanwhile, petroleum ether extracts are just as effective as aqueous methanolic extracts. It could be that the active ingredients in the fractions are relatively polar, considering that the A and B fractions have a better potency than the C fraction, taking into account the polarity of the solvent used in elution, the compounds may possess non-polar properties as well. In open-field tests, the total methanolic extract of *Z. majdae* and diazepam reduced movement, while the fractions did not. Aside from sedative effects, the fractions appear to suppress the tail flicks caused by withdrawal syndrome (31). 

Studies have shown that the aqueous methanol fractions of this plant contain flavonoid and terpenoid compounds. Flavonoids affect the opioid system, and terpenoids affect benzodiazepine receptors, therefore they are capable of reducing morphine tolerance, dependence, and withdrawal syndrome (31)(33). Linalool reduces glutamate release (28). It also regulates the GABAergic system and increases the affinity of GABA binding to GABA_A_ receptors (34). As *Z. majdae* has significant linalool content, a suggested mechanism for ZMEO effects against morphine dependence is by suppressing excitatory amino acids and affecting the GABAergic system, resulting in the reduction and reabsorption of glutamate (35). Another plausible mechanism is through dopaminergic pathways. Camphor and borneol (monoterpenes found in *Z. majdae*) inhibit the release of catecholamines such as dopamine due to the specific inhibition of the nicotinic acetylcholine receptors. Also, borneol inhibits norepinephrine that is induced by DMPP, thus reducing morphine tolerance and morphine dependence and improving the symptoms of the withdrawal syndrome. Camphor can also inhibit the increase of calcium induced by DMPP. By this mechanism, camphor reduces intracellular calcium concentration (29, 36). 

Another *in vivo* study goes through how the extracts from leaves of *Z. majdae* affect tolerance to the anticonvulsant activity of morphine and the withdrawal syndrome symptoms. In this study, morphine was administered subcutaneously at 2.5 mg/kg, twice a day for a total of four days with the last dose being administered before the PTZ test injection. On the day of the test, an hour before PTZ injection, ZMEO was administered at 20 and 40 mg/kg doses and it was significantly effective in decreasing the number of jumps in mice caused by the withdrawal syndrome. Another symptom of the syndrome is the excretion of dry stool which was only reduced when ZMEO was administered at a 40 mg/kg dose, thereby confirming that ZMEO is capable of reducing the various symptoms of the withdrawal syndrome. Co-treatment of 2.5 mg/kg of morphine and 20 mg/kg of ZMEO considerably lowered tolerance (37).

Linalool could play an important role in these effects, as mentioned in previous sections. In an *in vivo* study, linalool could inhibit the acquisition and reinstatement of morphine-induced conditioned place preference in mice. Doses of 12.5 and 50 mg/kg of linalool prevented the acquisition of morphine-induced conditioned place preference and doses of 25 and 50 mg/kg inhibited reinstatement of morphine-induced conditioned place preference. Linalool has an antagonistic effect on NMDA receptors. NMDA receptor antagonists can prevent the rewarding action of morphine by inhibiting NMDA receptors in the nucleus accumbens and ventral tegmental which are responsible for rewarding actions (38). Glutamate modulates dopamine which itself is an important neurotransmitter in the rewarding pathway (39). The euphoria induced by dopamine causes morphine reinforcement. Dopamine levels rise as a result of NMDA infusion into the nucleus accumbens. The glutamatergic pathway of the nucleus accumbens plays an important role in the regulation of the mesolimbic dopaminergic system and inducing opioid addiction (40, 41). 

The main constituent responsible for reducing morphine tolerance, dependence, and withdrawal syndrome symptoms is linalool. The antagonistic effect of linalool on NMDA receptors may also be relevant to its role in reducing morphine dependence and morphine tolerance. Linalool reduces the production or release of NO. As NO plays a significant role in the antinociceptive property of morphine, it is plausible that linalool follows the same mechanism. NO is also an intercellular messenger closely related to NMDA glutamate receptors. The effects of linalool are partially dependent on adenosine A_1_ and A_2A_ receptors. Adenosine A_1_ receptor agonists suppress withdrawal syndrome symptoms (42).

***Antidiabetic effects***

Diabetes is one of the major systemic diseases in the world. Alpha-amylase and alpha-glucosidase inhibitors are important in the treatment of diabetes (2). Alpha-glucosidase can break down carbohydrates into glucose. This enzyme can cause hyperglycemia in people with diabetes. Therefore, the inhibition of alpha-glucosidase is a way of treating hyperglycemia. The discovery of alpha-glucosidase inhibitors in natural sources has led to the production of safe and easily manufactured drugs replacing previous diabetes medications such as miglitol, voglibose, and acarbose (43).

In an *in vitro* study involving methanolic and aqueous extracts of the leaves of *Z. majdae*, the enzymatic activity of alpha-glucosidase was evaluated colorimetrically by observing the release of p-nitrophenol from p-nitrophenyl glycoside substrate in phosphate buffer. According to the study, *Z. majdae* extracts have inhibitory effects (50% and higher) on the alpha-glucosidase enzyme, making them a viable candidate for diabetes treatment. The methanolic extract showed 50–75% inhibition of alpha-glucosidase while the aqueous extract had less potent effects of about 25-50% inhibition (43).

In another *in vitro* study where acarbose was used as a positive control, the inhibitory effect of butanol and methanol extracts of *Z. majdae* parts including leaves, stems, flowers, and fruits on alpha-amylase enzyme were examined at different concentrations (15-30 mg/ml). The butanol and methanolic extracts can dose-dependently inhibit alpha-amylase activity. The highest inhibition of butanol extract was observed at a dose of 30 mg/ml by 77.9±2.1%, in comparison to acarbose which inhibited the alpha-amylase activity at a dose of 20 mg/ml by 73.9±1.9%. The butanol and methanolic extracts showed IC_50_ values of 24.5±2.1 and 22.0±2.7 mg/ml, respectively. Acarbose demonstrated an IC_50_ value of 6.6±3.1 mg/ml. Although both butanol and methanolic extracts can be useful in the treatment of diabetes, further *in vitro* and *in vivo *studies are necessary (2).

*Anti-oxidant effects*

Free radicals have detrimental effects on the human body due to their oxidative nature. Anti-oxidants are compounds that prevent these oxidative effects and are found to be useful in the treatment of some chronic diseases (21). Although lipid oxidation in food products can be prevented by Anti-oxidants, synthetic Anti-oxidants are not suitable choices due to their toxicity (44). Researchers are trying to find natural Anti-oxidant sources to better preserve food substances and reduce oxidative stress in living tissues (21)**.**

Due to the presence of some diterpenes such as labdane which exist in the roots of *Z. majdae, *this plant has Anti-oxidant properties (21). Labdane diterpenes can scavenge radicals, thus their Anti-oxidant activities. 12, 16-dideoxy aegyptinone B and 12-deoxy-salvipisone are diterpene quinones with rearranged abietane skeletons that were isolated from the roots of *Z. majdae* (6). Anti-oxidant activity can also be found in the extracts from the aerial parts which can be attributed to the phenolic and flavonoid content. In one *in vitro* study, the EO was extracted from the aerial parts of the *Z. majdae* plants in five areas of Iran (Sirmand, Ghotbabad, Sarchahan, Tangezagh, and Geno). They were evaluated for their anti-oxidant properties in the flowering stage using DPPH and β-carotene/linoleic acid assays. These tests were done at 1.25-40 mg/ml concentrations of ZMEO. Using DPPH, the most significant results were reported from Sirmand ZMEO, with an IC_50_ value of 8.01 mg/ml. In Ghotbabad ZMEO, an IC_50_ value of 8.79 mg/ml was observed. The least significant results were observed in Geno ZMEO, with an IC_50_ value of 18.47 mg/ml. The anti-oxidant effects of ZMEO in the Geno location were less potent than the effects of vitamin C and BCB with IC_50_ values of 0.009 and 0.013 ml/mg, respectively. Using β-carotene/linoleic acid assays, the IC_50_ values were ranging from 11.77 to 29.82 mg/ml. Similar to DPPH results, Sirmand ZMEO was the most effective (IC_50_ value of 11.77 mg/ml), the second Ghotbabad (IC_50_ value of 13.65 mg/ml), and the least effective were the samples tested in Geno ZMEO (IC_50_ value of 29.82 mg/ml). Under this evaluation, ZMEO potency was determined to be less than that of BHT and vitamin C (25).

Similar to the method used in the study above, the anti-oxidant activity of EO and various extracts have been analyzed. In the DPPH method, ZMEO and the methanolic extract showed IC_50_ values of 20.5±1.6 and 26.1±1.5 mg/ml, respectively. The effects were similar to the effects of BHT, a synthetic anti-oxidant. In the β-carotene/linoleic acid method, ZMEO and the methanolic extract showed 19.2 and 25.8 mm mean zones of color retention, respectively. No activity was observed in the other types of extracts. The methanolic extract contained the greatest amount of phenolic compounds (50.1±2.3 μg/mg), with a flavonoid content of 48.4 μg/mg. The high anti-oxidant capacity may be attributed to the presence of such compounds (21).

An *in vivo* study examined the effect of linalool on neurotoxicity induced by acrylamide (ACR) in rats. IP injection of 12.5, 25, 50, and 100 mg/kg doses of linalool were evaluated in rats in three different groups; The first group of rats received an injection three days before IP injection of ACR (50 mg/kg), another received simultaneous with ACR (50 mg/kg) IP administration, and the third group received injection three days after ACR (50 mg/kg) IP administration. Then, the levels of MDA as a marker of lipid peroxidation and GSH were measured in the brain tissue of rats. GSH protects cells against reactive oxygen species. Exposure to ACR is known to decrease GSH levels in the cerebral cortex and cause the brain tissues to become more sensitive to oxidative stress. Following this raised sensitivity, an increase in MDA level occurs leading to lipid peroxidation. It is plausible that anti-oxidants can be used in the modulation of ACR-induced toxicity. Linalool, an example of an anti-oxidant, dramatically inhibited DMBA-induced lipid peroxidation in female rats. Results showed that administration of linalool decreased neurotoxicity and gait abnormality induced by ACR, especially at a 12.5 mg/kg dose, this is due to linalool increasing the level of GSH in the cerebral cortex and decreasing the level of MDA. In this study, vitamin E was used as positive control and its effect was similar to 12.5 mg/kg of linalool. Aside from the dosage, the timing of administering linalool was shown to be important as well. Administration three days after treatment with ACR proved to be ineffective (45).

In an *in vitro* study, the association between the number of phenolic compounds and anti-oxidant activities of butanol fraction and ethyl acetate subfractions of *Z. majdae* has been evaluated. The radical scavenging effects and reducing power were studied to determine the anti-oxidant activity of *Z. majdae*. The ethyl acetate fraction was produced using liquid-liquid extraction. Further fractionation produced 5 subfractions using MPLC. Among these five subfractions, subfraction 2 showed the most potent inhibitory effect on radical activity in the radical scavenging assay (IC_50_ value=41.85±0.61 μg/ml). The effect is similar to the inhibitory effect of quercetin. It was also observed that ethyl acetate subfraction 2 has the greatest amount of phenolic compounds (1.98±0.01 mg/g) by using Folin-Ciocalteu reagent and flavonoid contents (357.4±18.7 μg/ml) and aluminum chloride colorimetric assay. The ethyl acetate fraction showed the lowest amount of phenolics which was 0.98±0.095 mg/g. The butanol fraction demonstrated the lowest amount of flavonoids which was 1.2±0.006 µg/ml (44).

In the reducing power assay, the reducing power of quercetin was higher than the other subfractions. The ethyl acetate fraction showed higher reducing power than other subfractions. No relationship was found between the inhibitory effect on radical activity and phenolic and flavonoid content. The flavonoids may be phenolic types of *Z. majdae* subfractions that display anti-oxidant activity (44). These results conclude the usefulness of *Z. majdae *in the pharmaceutical, perfume, and food industries.

*Toxicity*

An *in vivo* study evaluated the acute toxicity of aqueous infusion and the ethanolic extracts of the aerial parts of *Z. majdae* in mice. Different doses of these extracts were IP injected into mice. After 48 hr, the mortality rate was measured. The aqueous infusion showed an LD_50_ value of 3.09 g/kg body weight and the ethanolic extracts showed an LD_50_ value of 3.94 g/kg body weight. Therefore, the ethanolic extract was less toxic than the aqueous infusion, meanwhile, both of these are moderately toxic. The aqueous infusion and the ethanolic extracts were most effective at preventing death at the doses of 2 g/kg body wt. and 2.8 g/kg body wt., respectively (8).

In another *in vivo* study, the neurotoxicity (movement toxicity and sedation) of ZMEO and methanolic extract of aerial parts of *Z. majdae* has been investigated using the rotarod performance test in mice. Different doses were injected into mice. After 24 hr, the number of deaths was counted. ZMEO showed an LD_50_ of 2.35 (1.98–2.65) ml/kg and methanol extract showed 34% mortality at the dose of 5 g/kg. For the neurotoxicity experiment, the doses of 3 and 4 g/kg of methanol extracts, 1.5, 1.75, and 2 ml/kg of ZMEO, and 0.1 ml/10 g of the solvents (sesame oil and tween 80 (5 % v/v)) were injected. In the rotarod test, the doses of 0.65 ml/kg of ZMEO and doses of 2 g/kg of the methanolic extract reduced the walking time. Neurotoxicity was dose-dependent and EO showed sedation and motor deficit at TD_50_ of 0.55 ml/kg. Sedation occurred due to the presence of some terpenes including linalool. Also, camphor may be responsible for the lethality of ZMEO (27).

An *in vivo* study on the effects of *Z. majdae* on *Pangasianodon hypophthalmus*, or catfish, evaluated growth, immunology, hematology factors, and toxicity. The fish were separated into four groups based on the dose of extract they received. The control group received food without any drugs, while the other three groups received 150, 300, and 600 mg of the drug per every kilogram of their food intake for 45 days. According to the results, those fish that received the extracts with their food showed a survival rate of 100%. Further study of the efficiency is necessary to observe the impacts of transport and crowding stress, temperature variation, and the presence of an uncontrolled bacterial population (46). More toxicological studies such as testing for teratogenicity are required in this area to further elaborate on the toxicity of the extracts and the constituents of *Z. majdae*.

**Table 1:** Review of pharmacological effects of *Zhumeria majdae*

**Effect**	**Study design**	**Part of plant/constituent** **Preparation**	**Result/mechanism**	**References**
Anti-inflammatory	*In vivo*	Aerial parts/Aqueous infusion and ethanolic maceration extracts	Reduce capillary permeability	(8)
	*In vivo*	Aerial parts/ Methanolic extracts	Reduce edema induced by carrageenan	(9)
	*In vivo*	Leaves/ EO	Reduce NO activity, inhibit prostaglandin pathway	(10)
Antinociceptive	*In vivo*	Leaves/ EO	Block TRPA1 receptor, inhibit prostaglandin pathway	(10)
	*In vivo*	Aerial parts/ EO, aqueous and ethanolic extracts	Central opioid agonist	(8)
	Hot plate test in mice	Aerial parts/ Methanol extracts	Increase pain threshold and tolerance	(9)
Antifungal-* A. flavus, M. canis, M. gypseum, T. rubrum, E. floccosum, C. albicans, T. mentagrophytes*	Punch well/cup plate diffusion method	Aerial parts/ Hydro-distilled oil	Inhibition zone = 7.84 - 29.05 mM Average MIC value = 0.015 μl/ml	(4)
*A. niger, C. albicans, A. flavus*	*In vitro*	Aerial parts/ EO	*A. niger*: MIC= 0.5 μl/ml MBC=8 μl/ml *A. flavus*: MIC=2 μl/ml MBC=8 μl/ml *C. albicans*: MIC=2 μl/ml MBC=4 μl/ml	(13)
*S. cerevisiae, A. niger, C. albicans*	*In vitro*	Aerial parts/ EO	*S. cerevisiae* is most sensitive with an inhibition zone of 16 mm and MIC of 5 mg/ml	(14)
*F. graminearum, F. asiaticum, F. redo- lens f.sp. dianthus, F. verticillioides, F. oxysporum f.sp. lentis, S. sclerotiorum, A. flavus, A. tubingensis, B. cinerea, C. cladosporioides*	*In vitro*	Aerial parts/ EO	Full potency against *F. graminearum, F. asiaticum, S. sclerotiorum*	(15)
*C. albicans and A. niger*	*In vitro*	Aerial parts/ EFEO and EO	EFEO is more effective against *C. albicans* than EO, EFEO, and EO equally effective against *A. niger*	(16)
Antityrosinase	*In vitro*	Leaves/ Hydroalcoholic extract	Inhibit mushroom tyrosinase, inhibit l-DOPA oxidation	(17)
Antiprotozoal- *Trypanosoma brucei, Leishmania donovani, T. cruzi, T. brucei rhodesiense, Plasmodium falciparum*	*In vitro*	Root and aerial parts/ Hexane, ethyl acetate, and methanol extracts	Root parts most potent, hexane extract of the roots was highly effective against *T. b. Rhodesiense*, *P. falciparum*, and *L. donovani*	(18)
*L. donovani, P. falciparum*	*In vitro*	Root/ Methanol extracts	IC_50_ for *L. donovani*= 1.1 μg/ml, IC_50 _for chloroquine-sensitive strains of *P. falciparum*= 8.8 μg/ml and IC_50 _for chloroquine-resistant strains= 7.5 μg/m	(19)
Antibacterial- *Helicobacter pylori*	*In vitro*	Aerial parts/ Methanol extract, chloroform extracts, and EO	EO is more effective against *H. pylori* than methanol and chloroform extracts	(20)
*Bacillus subtilis, Enterococcus faecalis, Staphylococcus aureus, S. epidermidis, Escherichia coli, Pseudomonas aeruginos, Klebsiella pneumoniae*	*In vitro*	Aerial parts/ EO	High inhibition of Gram-positive bacteria Low to moderate inhibition of Gram-negative bacteria	(14)
*S. aureus, E. coli, K. pneumoniae, S. epidermidis, B. subtilis, P. aeruginosa*	*In vitro*	Aerial parts/ EO, the aqueous and methanol extracts	EO at 0.39 mg/ml showed notable antibacterial activity	(21)
*S. aureus, S. epidermidis, S. saprophyticus, L. monocytogenes, B. cereus, B. subtilis, E. coli, S. typhi, P. aeruginosa, S. dysenteriae, S. flexneri, K. pneumoniae, P. vulgaris, E. aerogenes, E. faecalis, B. pumilus*	*In vitro*	Aerial parts/ EO	Bactericidal activity against *S. aureus, B. cereus, E. coli*, *E. aerugenes, *inhibitory effect against *B. subtilis* and *P. vulgaris*.	(13)
*E. coli and S. aureus*	*In vitro*	Aerial parts/ Encapsulation of ZMEO by lecithin	EO and EFEO showed equal MIC_50_ and MIC_90 _values of 0.6% and 1.25%, respectively.	(16)
Antibiotic-sensitive strain of *E. coli* (Dh5α)	*In vitro*	Aerial parts/ EO	Inhibition of Dh5α growth	(22)
larvicidal- *Anopheles stephensi, Culex quinquefasciatus*	*In vitro*	Leaves/ EO	*An. stephensi* LC_50_=61.3 ppm, LC_90_=135.8 ppm; *Cx. quinquefasciatus* LC_50_=88.51 ppm, LC_90_=191.56 ppm.	(24)
Antiviral- *Herpes simplex type 1*	*In vitro*	Aerial parts/ Methanolic extract	High anti-HSV-1 effect at low concentrations	(23)
Anticonvulsant	*In vivo*, PTZ and MES models in mice	Leaves/ EO	Reduce frequency of PTZ clonic convulsions, increase chronic seizure threshold, the lower mortality rate	(11)
	*In vivo*, PTZ and MES models in mice	Aerial parts/ EO and methanolic extracts	Protecting from tonic convulsions in PTZ and MES Increase the onset of tonic convulsions in PTZ	(27)
Antidiabetic	*In vitro*	Aerial parts including leaves, stems, flowers, and fruits/ Butanol and methanol extracts	Inhibit alpha-amylase enzyme	(2)
	*In vitro*	Leaves/ Methanol and aqueous extracts	Inhibit alpha-glucosidase enzyme	(43)
Antioxidant	*In vitro*	Root/ Dried powdered	Scavenge radicals by labdane diterpenes	(6)
	*In vitro*	Aerial parts/ EO	IC_50_ value of 8.01 mg/ml	(25)
	*In vitro*	Air-dried and ground herbal parts and top flowerings of the plant/ EO and methanol extract	EO IC_50_ value=20.5 ± 1.6 Methanolic extract IC_50_ value=26.1 ± 1.5	(21)
	*In vitro*	Aerial parts/ Butanol fraction and ethyl acetate (sub) fractions	IC_50_ value= 41.85 ± 0.61 μg/ml	(44)
	*In vivo*	Linalool -	Inhibit DMBA-induced lipid peroxidation Modulation of ACR-induced toxicity and gait abnormality Increase GSH Decrease MDA	(45)
Cytotoxicity- human malignant melanoma (SK-MEL), human epidermal carcinoma (strain KB), human ductal carcinoma (BT549), human ovary carcinoma (SK-OV3), monkey kidney fibroblast (Vero), mouse macrophages (RAW 264.7)	*In vitro*	Root/ Methanolic extracts	Both the extract and the diterpene compound (dideoxy aegyptinone B-12, 16) show cytotoxicity	(19)
Human melanoma cell line (A375), human breast cancer cell line (MCF7)	*In vitro*	Aerial parts/ EO	Dose-dependent inhibition of cell growth Moderate cytotoxicity	(25)
Human breast adenocarcinoma (MCF7), Human cervix epithelial carcinoma (HeLa)	*In vitro*	Root/ Hexane extract	Great binding affinity to HSP90 protein Anti-tumor effect of lanugon Q in MCF7 with IC_50_ =20 ± 2 μM	(26)
Toxicity	*In vivo*	Aerial parts/ Aqueous infusion and ethanolic maceration extracts	Moderately toxic LD_50_ for the aqueous infusion= 3.09 g/kg body weight, LD_50_ for the ethanolic extracts = 3.94 g/kg body weight	(8)
	*In vivo*	Stem and leaves/ *Z. majdae* extract	Non-toxic in *Pangasianodon hypophthalmus* (catfish)	(46)
Neurotoxicity	*In vivo*	Aerial parts/ EO and methanolic extract	LD_50_ = 2.35 ml/kg TD_50_ = 0.55 mL/kg The mortality rate at the dose of 5 g/kg= 34%.	(27)
Effect on dependence, tolerance, and Withdrawal syndrome symptoms of morphine	*In vivo*	Leaves/ EO	Inhibit NMDA receptors, reduce NO, agonistic activity on adenosine receptor	(30)
	*In vivo*	Aerial parts/ Aqueous methanol fractions	Affect opioid receptors, reduce glutamate release, increase the affinity of GABA binding to GABA_A_ receptors, inhibit the release of catecholamines, inhibit norepinephrine, and reduces intracellular calcium concentration	(28,29,34,36)
	*In vivo*	Linalool/ -	NMDA receptor blockade, NO signaling, and adenosine receptor stimulation	(47)
	*In vivo*	Aerial parts/ Total extract and aqueous methanolic extract	Reduce withdrawal syndrome symptoms by affecting opioid receptors Partial agonist on benzodiazepine receptors	(31)
	*In vivo*	Leaves/ EO	Decrease withdrawal syndrome symptoms, reduce tolerance to the anticonvulsant effects of morphine	(37)
Effect of linalool on the acquisition and reinstatement of morphine-induced conditioned place preference	*In vivo*	Linalool/ -	Prevent the rewarding action of morphine by inhibiting NMDA receptors	(41)

**Figure 2:**
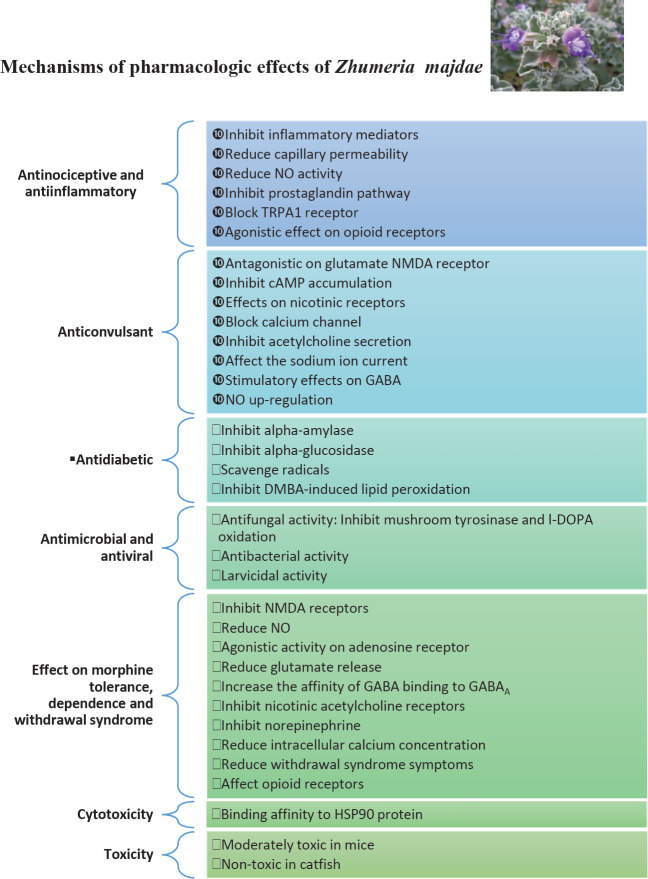
Summary of the mechanisms of the pharmacological effects of *Zhumeria majdae*

## Conclusion

In this review, different studies regarding the pharmacological effects of *Z. majdae* have been discussed. *Z. majdae* contains pharmacologically significant constituents with anti-inflammatory, antinociceptive, antimicrobial, antiviral, anticonvulsant, and anti-oxidant properties. For an overview of the effects refer to Table 1. It is also found to be useful in treating diabetes, reducing morphine tolerance and dependence, decreasing withdrawal syndrome symptoms, and for its cytotoxic properties. The majority of the studies are related to the antimicrobial effects of *Z. majdae*. The antifungal effect is caused by inhibition of the fungal tyrosinase enzyme. *Z. majdae* contains significant amounts of linalool which could be responsible for the antimicrobial activity by protein denaturation and solvent dehydration. Also, the presence of camphor, terpenes, and flavonoids could be an important factor in antimicrobial activity. Therefore, *Z. majdae* may be considered a valuable pharmaceutical compound with additional uses in other areas such as cosmetics and agriculture. Further research is needed to elaborate on the precise mechanism for the antimicrobial effects of *Z. majdae*. Based on current research, the antimicrobial effects of *Z. majdae* are similar to levofloxacin, clarithromycin, and metronidazole, with the antileishmanial effects being more potent than pentamidine.

A couple of studies have evaluated the anti-inflammatory and analgesic properties of *Z. majdae*. The anti-inflammatory effects are similar to the effects of dexamethasone, baclofen, and diclofenac. The analgesic effects are also similar to diclofenac. The mechanism of the anti-inflammatory activity consists of the inhibition of inflammatory mediators release and consequently a decrease in capillary permeability, reduced NO activity, and inhibition of prostaglandins. The analgesic effects are caused by a decrease in voltage-dependent Na^+^ flow in ganglion cells, inhibition of prostaglandin synthesis through the cyclooxygenase pathway, and the induction of endogenous opioid peptide release by affecting central opioid receptors. The extracts of *Z. majdae* have also shown antiviral effects by blocking the surface ligands and receptors of the virus or the host. The extracts also demonstrate anticonvulsant properties similar to diazepam. These properties are due to the antagonistic effect on glutamate NMDA, along with inhibiting intracellular cAMP accumulation, effects on nicotinic receptors, calcium channel blocking, and inhibiting acetylcholine secretion. The extracts also affect voltage-dependent sodium ion channels and have a stimulatory effect on GABA_A_ receptors as well as cause NO up-regulation and protect the central nervous system from seizures.

*Z. majdae* also shows inhibitory activity against alpha-glucosidase and alpha-amylase enzymes, making it a possible candidate for diabetes treatment. There are studies on the anti-oxidant and cytotoxic effects, however, more research is necessary for understanding the underlying mechanisms, especially how the effects vary *in vitro *compared with *in vivo *studies. 

There is significant evidence for effects against morphine dependence, morphine tolerance, and withdrawal syndrome. The potency of *Z. majdae* in these cases is similar to clonidine and diazepam. Several factors such as adenosine, the adrenergic system, excitatory amino acids, and PKC inhibitors can affect withdrawal symptoms. For a compound to suppress withdrawal symptoms, it must be able to act on several different factors. *Z. majdae* constituents are partial agonists for benzodiazepine receptors and are therefore capable of reducing morphine withdrawal symptoms. Linalool, a main constituent of the extracts, regulates glutamate activity and reduces glutamate release, (as a competitive antagonist for L-glutamate). Linalool also regulates the GABAergic system and increases the affinity of GABA binding to GABA_A_ receptors. As *Z. majdae* has significant linalool content, a suggested mechanism for the effects against morphine dependence is through suppressing excitatory amino acids and affecting the GABAergic system, resulting in reduction and reabsorption of glutamate. Linalool affects the release of NO which itself is a messenger closely related to NMDA glutamate receptors. Linalool is also an agonist of adenosine A_1_ and A_2_ receptors. Adenosine A_1_ receptors are important in suppressing withdrawal syndrome symptoms. Through its agonist effects on adenosine A_1_ and its role in reducing NO release, linalool may suppress withdrawal syndrome symptoms. Two of the constituents of *Z. majdae*, camphor, and borneol, can inhibit dopamine release. Borneol also inhibits norepinephrine. These lead to reduced morphine tolerance and morphine dependence, improving withdrawal syndrome symptoms. Camphor is also capable of reducing intracellular calcium concentration. It also appears that *Z. majdae *extracts reduce tolerance to the antinociceptive effects of morphine by affecting the opioid system and opioid receptors. For a summary of the mechanisms see Figure 2.

In this article, we also reviewed the toxicity of *Z. majdae* and its main constituent, linalool. *Z. majdae* has been observed to be non-toxic in catfish, however, it showed moderate toxicity in mice. Despite this fact, linalool alone reduces acrylamide-induced neurotoxicity in mice.

There is a significant shortage of clinical evidence on various effects of the extracts of *Z. majdae*. Clinical studies are required to confirm the pharmacological properties.

## Authors’ Contributions

KK and AM wrote the manuscript. HH designed and revised the review. All the authors read and approved the final manuscript.

## Conflicts of Interest

The authors have no conflicts of interest to declare.
